# Multiomics Profiling Supports Cathepsin B as a Protective Factor in Cerebral Small Vessel Disease

**DOI:** 10.21203/rs.3.rs-9013964/v1

**Published:** 2026-04-19

**Authors:** Cyprien Rivier, Sebastian Diaz-Perez, Gael Bories, Shufan Huo, Santiago Clocchiatti-Tuozzo, Adam de Havenon, Stephanie Debette, Kevin Sheth, Lauren Sansing, Guido Falcone

**Affiliations:** Yale School of Medicine; Yale; Yale; Yale School of Medicine; Yale; Yale; University of Bordeaux; Yale University; Yale University; Yale School of Medicine

## Abstract

White matter hyperintensities (WMH) are a common brain MRI marker of cerebral small vessel disease and a major risk factor for stroke and dementia, yet the molecular mechanisms governing lesion formation remain incompletely understood. Here, we combined large-scale proteomic and genomic data with tissue- and single-nucleus transcriptomic validation to identify circulating proteins that influence WMH volume and its downstream clinical manifestations. Association and Mendelian Randomization analyses of WMH volume and 2,923 plasma proteins in 52,560 UK Biobank participants identified the lysosomal protease cathepsin B (CTSB) and the mitochondrial enzyme Enoyl-CoA Hydratase Domain Containing 3 (ECHDC3) as the top candidates associated with WMH volume. Single-nucleus RNA sequencing of post-mortem periventricular white matter from 8 individuals (4 with WMH lesions and 4 matched controls) revealed a marked downregulation of CTSB in WMH lesions, predominantly in astrocytes and oligodendrocytes, whereas ECHDC3 expression was unchanged between cases and controls. In the UK Biobank, higher plasma CTSB levels and a cis-pQTL polygenic score each predicted better general cognitive ability, independent of vascular risk factors, whereas ECHDC3 showed no cognitive association. Together, these multimodal analyses across different studies point to CTSB as a lesion-suppressed, cognition-enhancing factor and a plausible therapeutic target for mitigating the effects of WMH and preserving brain health.

## INTRODUCTION

Cerebral small vessel disease (CSVD) is increasingly recognized as a major contributor to poor brain health^1^. CSVD represents a highly prevalent group of diseases that compromise the cerebral small vessels of the brain^2^. CSVD is a major risk factor for several conditions driving poor brain health, including small vessel ischemic stroke, spontaneous intracerebral hemorrhage, cognitive decline and dementia, parkinsonism and late-life depression^3–5^. A number of therapeutic interventions focused on upstream risk factors are known to improve or delay CSVD, including aggressive blood pressure and diabetes management and optimization of healthy habits (exercise, diet and sleep)^6^; however, treatments squarely focused on CSVD are still lacking.

Research focused on CSVD has relied heavily on neuroimaging markers that allow investigations in asymptomatic persons. White matter hyperintensities (WMH) constitute one of the most widely used of these biomarkers^7^. WMH are white matter lesions appearing hyperintense in FLAIR and T2 sequences of brain MRIs and are found in the majority of individuals over 60 years of age, with prevalence estimates ranging from approximately 45% to over 95% depending on the population studied^8^. Important advantages of WMH include easy and standardized ascertainment, significant accumulated experience and, most importantly, strong links with clinical entities linked to CSVD. WMH precede cognitive decline, with greater burden or polygenic risk being associated with accelerated conversion of mild cognitive impairment to dementia^9,10^. Convergent genetic, Mendelian randomization and autopsy studies demonstrate that arteriolosclerosis, myelin rarefaction and hemodynamic stress, all drive WMH lesion formation, highlighting the heterogeneity of the phenotype^11,12^. Despite the compelling evidence supporting WMH burden as an upstream causal determinant of several clinical endpoints, there are no existing treatments that specifically target this CSVD manifestation.

Recent work has moved beyond describing clinical or imaging associations to dissect the molecular pathways by which vascular stress injures white matter. Integrative genome-wide association study (GWAS), epigenomic and proteomic analyses implicate diverse loci that map to vascular, astrocytic and oligodendroglial biology, with convergence on immune signaling and myelin maintenance pathways^13,14^. However, translating these association signals into actionable mechanisms remains challenging. Circulating proteins are attractive as mechanistic probes and therapeutic entry points, yet many protein signals are pleiotropic, context-dependent across tissues, and may represent downstream responses to injury rather than upstream drivers of lesion formation. Consequently, candidate-based follow-up can conflate causal effectors with biomarkers that track disease severity or compensation. A systematic analytical framework is therefore required to distinguish proteins that lie upstream of WMH from those reflecting secondary, indirect, or reactive processes.

**Figure 1:**
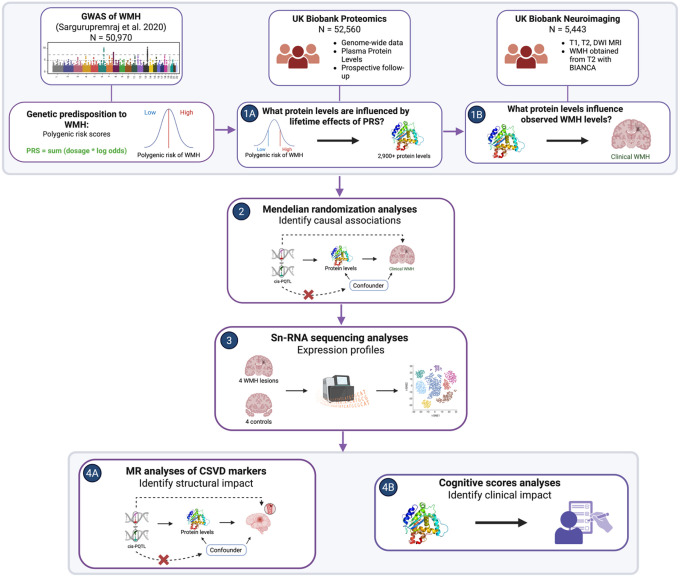
Graphical abstract Graphical abstract. Overview of the 4-stage proteogenomic framework used to prioritize circulating proteins implicated in WMH. A WMH GWAS was used to derive a WMH polygenic risk score, which was integrated with plasma proteomics from the UK Biobank Proteomics (UKB-PPP; N=52,560; >2,900 proteins) and brain MRI-derived WMH from the UK Biobank neuroimaging subset (N=5,443). Step 1A identified plasma proteins associated with lifetime genetic predisposition to WMH, and Step 1B evaluated associations between measured protein levels and observed WMH burden. Step 2 applied cis-pQTL–based Mendelian randomization to test protein-WMH causal effects while mitigating confounding. Step 3 assessed expression profiles of prioritized targets using snRNA sequencing from WMH lesions and controls (four lesions, four controls). Step 4 extended analyses to additional CSVD imaging markers (4A) and cognitive outcomes (4B) to contextualize structural and clinical relevance.

To meet this need, we devised a conservative, unbiased, four-stage proteogenomic study (Fig. 1). Stage 1 couples a WMH polygenic risk score (PRS) with high-throughput plasma proteomics in > 50 000 UK Biobank participants, identifying proteins where inherited risk converges^15^. Stage 2 applies bidirectional Mendelian randomization, employing cis-protein quantitative trait loci (pQTL) and cis-expression quantitative trait loci (eQTL) instruments to test causality in WMH and related clinical traits. Stage 3 interrogates single-nucleus RNA-seq data from WMH lesions to determine whether the candidate proteins are transcriptionally perturbed at the site of injury. Stage 4 examines both circulating and genetically predicted protein levels in relation to multidimensional cognitive performance in patients enrolled in the UK Biobank. This integrated approach triangulates multimodal data from different studies and allows for the discovery of mechanistic effectors and their potential therapeutic leverage points. Since each stage applies an independent and orthogonal filter, signals that survive all four steps are likely to represent true biological relationships rather than statistical artifacts.

In this study, we show that cathepsin B (CTSB), a lysosomal protease, withstands this multi-layered interrogation. In the UK Biobank, CTSB levels and cis-pQTL and cis-eQTL instruments consistently associate with lower WMH volume, and reverse analyses confirm the directionality of effect. Across vascular and brain-specific eQTLs, the protective effect of CTSB is consistent, reinforcing a model in which it operates both systemically and within glia to buffer microvascular stress. Lesion-level single-nucleus transcriptomics reveal pronounced downregulation of CTSB, most notably in astrocytes and oligodendrocytes, consistent with the pattern predicted by our genetic analyses. Higher plasma CTSB levels and a CTSB polygenic score further associate with superior cognitive performance. Collectively, these findings nominate CTSB as a promising molecular lever in WMH pathophysiology and suggest a therapeutic strategy focused on augmenting CTSB activity.

## RESULTS

We carried out a proteomic study in the UK Biobank-Pharma Proteomics Project (UKB-PPP), where plasma proteomic profiling was done with the Olink Explore 1536 and Explore Expansion platform, targeting 2,923 unique proteins. In a four-stage analysis, we first identified CTSB and Enoyl-CoA hydratase domain-containing protein 3 (ECHDC3) as candidate plasma proteins potentially mediating the polygenic risk of WMH (Stage 1). Subsequently, we assessed the involvement of these proteins as drivers of WMH pathology using bidirectional Mendelian Randomization (MR) analyses (Stage 2). Then, we assessed differential expression of CTSB and ECHDC3 within WMH lesions compared to control tissues using single-nucleus RNA sequencing (Stage 3). Finally, to investigate the involvement of CTSB and ECHDC3 beyond WMH, we explored their association with related cerebrovascular traits and cognitive performance (Stage 4).

### Stage 1: Associations between polygenic risk, WMH volume, and protein levels

First, we derived a PRS from 27 independent genetic variants (Table S1) identified in the most recent GWAS of WMH^16^ in a cohort of 52,560 individuals (mean age: 56.8, 53.9% female sex, Table S2) with genetic data and protein level measurements. The median number of participants having data for a given protein level was 44,480 (interquartile range: 43,434–50,998). The association between the polygenic risk for WMH (independent variable) and each protein level (dependent variable) was evaluated using linear regression models adjusted for age, sex, and the first 10 genetic principal components. With this approach, we identified eight proteins whose levels differed significantly with respect to polygenic risk of WMH (modeled as a continuous variable) at a false discovery rate (FDR) of 5%. Of these, five were positive associations (ECHDC3, LRRC37A2, NID2, ESM1 and BCAM), while three were negative associations (CTSB, GYS1, S100A14, Figure S1, Table S3).

**Figure 2:**
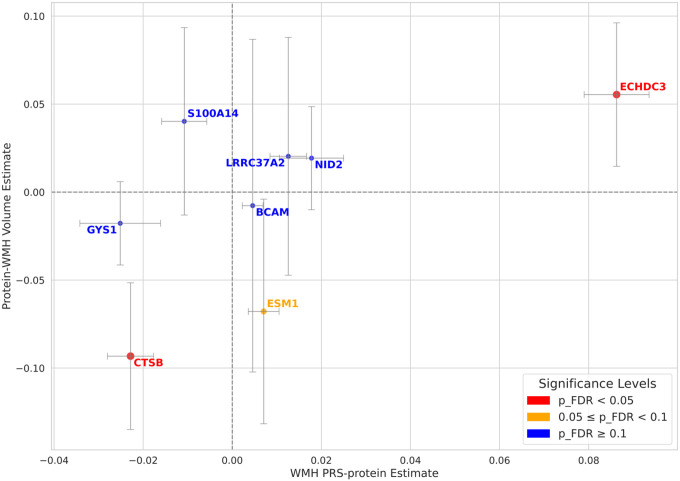
Associations of protein levels with a PRS of WMH and WMH volume Scatter plot showing the joint associations of 8 proteins with (1) a PRS for WMH on the x-axis and (2) radiographic WMH volume on the y-axis. The 8 proteins were selected out of 2,923 because they were significantly associated with the PRS at an FDR of 5%. The x-axis (WMH PRS–protein estimate) represents the beta effect size from linear regression models adjusted for age, sex, and the first 10 genetic principal components. The y-axis (protein– WMH volume estimate) reflects the beta effect size from analogous regressions of WMH volume on protein levels. Proteins are colored according to the FDR-adjusted significance of their association with WMH volume. Error bars represent 95% confidence intervals. Source data are provided as a Source Data file.

Second, we tested for associations between these eight identified proteins and WMH volume. WMH volume was ascertained using brain MRIs at a median of 9.3 years (Q1–Q3: 7.8–10.4) after the baseline blood sample collection used for the proteomic and genomic analyses. The subset of UKB-PPP participants who also took part in the neuroimaging UKB study comprised 5,443 participants (mean age: 54.2, 53.5% female sex, Table S4). The association between each protein (independent variable) and WMH volume (dependent variable) was evaluated using linear regression models adjusting for age, sex, and the first four genetic principal components. We identified two of the eight proteins significantly associated with WMH volume at an FDR of 5%: ECHDC3 and CTSB (Fig. 2, Table S5). Furthermore, beyond statistical significance, we note that both proteins had concordant directionality of effects between their associations with the PRS and their associations with WMH volume: ECHDC3 was associated with higher levels of the PRS (beta = 0.09, se = 0.003, p < 10^−118^) and higher WMH volume (beta = 0.06, se = 0.015, p < 10^−4^) while CTSB was associated with lower levels of the PRS (beta = −0.02, se = 0.002, p < 10^−17^) and lower WMH volume (beta = −0.09, se = 0.023, p < 10^−4^). Additionally, out of the 27 loci identified for WMH volume and included in our PRS, one was located in cis-position (+/− 1Mb of the gene) for CTSB (rs7004825) and one was located in cis-position for ECHDC3 (rs11257311), confirming the involvement of these genes in the genetic architecture of WMH.

### Stage 2: Mendelian Randomization

**Figure 3:**
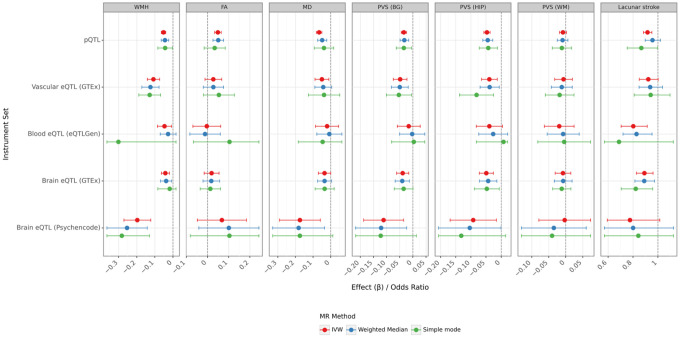
Mendelian Randomization Results of CTSB expression on CSVD traits. Forest plot of Mendelian randomization estimates and 95% confidence intervals for the protein CTSB on seven markers of cerebral small vessel disease: white matter hyperintensities (WMH), fractional anisotropy (FA), mean diffusivity (MD), perivascular spaces in the basal ganglia (PVS (BG)), hippocampus (PVS (HIP)), and white matter (PVS (WM)), and lacunar stroke. The analysis uses five distinct instrument sets: pQTL, vascular eQTL (GTEx), blood eQTL (eQTLGen), brain eQTL (GTEx), and brain eQTL (PsychEncode). Within each panel, point estimates are colored by the MR method used: inverse-variance weighted (IVW; red), weighted median (blue), and simple mode (green). The horizontal bars represent the 95% confidence intervals. The dashed vertical line indicates the null effect (an effect of β=0 for the first six outcomes and an Odds Ratio of 1 for lacunar stroke). Source data are provided as a Source Data file.

We performed bidirectional MR to test whether ECHDC3 and CTSB were causally associated with changes in WMH volume. This approach also allowed us to test for the directionality of these effects to ensure that the protein is influencing the outcome and not vice versa. For this purpose, we leveraged several sets of genetic instruments known to influence the transcription and circulating protein levels of ECHDC3 and CTSB. We used eQTLs to model the mRNA expression of ECHDC3 and CTSB, and pQTLs to genetically model the circulating levels of the protein products of these genes. Since vascular and cerebrovascular diseases are important contributors to WMH, we identified a set of eQTL instruments obtained in whole blood (eQTLGen^17^) and another set obtained from vascular tissues (GTEx^18^). In addition, to capture the possibility that the proteins of interest represent a response of the brain tissues to initial vascular injury, we also included two sets of eQTL instruments obtained exclusively from brain tissue (GTEx and PsychEncode^19^). All these instruments were derived from studies performed in individuals of European ancestry. We observed robust and consistent results of the effect of CTSB expression on WMH volume, with all 5 sets of instruments indicating that a higher mRNA expression or protein levels of CTSB were associated with lower WMH volume (Fig. 3, Table S6). The directionality of the causal effect was confirmed when running reverse MR analyses: there was no significant effect of WMH volume on CTSB expression (Figure S3, Table S7). In combination, these results suggest that CTSB lies upstream of WMH in the causal pathway and mediates part of the polygenic effect on WMH volume. We did not observe such associations between ECHDC3 expression and WMH volume (Figure S2, Table S8).

### Stage 3: Single-nucleus expression analyses of CTSB and ECHDC3

**Figure 4:**
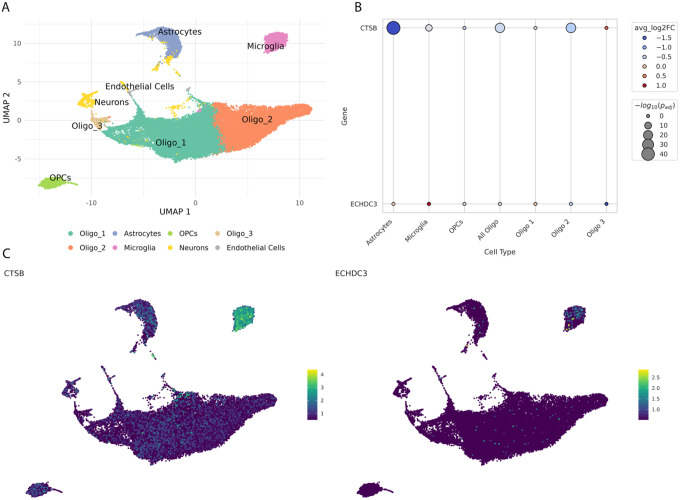
Differential Expression in snRNA of periventricular WMH lesions. A. UMAP plot of all nuclei from the snRNA-seq analysis. Each dot represents a single nucleus; colors denote cell-type clusters (see legend). B. Dot plot showing differential expression of CTSB and ECHDC3 in snRNA-seq data from 4 periventricular WMH lesions versus 4 controls across seven central nervous system cell types: astrocytes, microglia, oligodendrocyte precursor cells, all oligodendrocytes, and three oligodendrocyte subtypes. Within the plot, each point represents a single gene–cell-type comparison. Color denotes the average log2 fold change (red indicates upregulation in WMH lesions relative to controls; blue indicates downregulation), and size denotes statistical significance (−log10 adjusted p-value). Source data are provided as a Source Data file. C. UMAP feature plots showing log-normalized expression of CTSB (left) and ECHDC3 (right) across all clusters shown in Panel A.

To establish whether CTSB and ECHDC3 are perturbed in WMH lesions, we analyzed single-nucleus RNA-seq data from an ongoing, prospective study focused on understanding the pathophysiology of CSVD leveraging human brains. Specifically, we analyzed data from eight post-mortem periventricular white matter samples: four with WMH lesions and four age- and sex-matched controls (Table S10). Unsupervised clustering resolved the major CNS cell lineages—astrocytes, microglia, oligodendrocytes, oligodendrocyte-precursor cells, neurons and endothelial cells—and, as expected for white matter^20^, oligodendrocytes predominated, whereas neurons and endothelial cells were scarce (Fig. 4A). Cell-type proportions did not differ significantly between WMH and control tissue, indicating that subsequent transcriptomic differences reflect true molecular dysregulation rather than compositional shifts (Figure S5, Table S12). Differential single-nucleus expression analyses revealed a strong, cell-type-specific suppression of CTSB transcripts within lesions. The largest effect was seen in astrocytes (log FC = − 1.7, FDR p- value < 1 × 10 ^42^), followed by oligodendrocytes (log FC = − 0.48, FDR p-value < 1 × 10 ^20^) and microglia (log FC = − 0.35, FDR p-value < 1 × 10 ^8^, Table S13). In contrast, ECHDC3 showed only modest absolute changes (-−0.2 < log FC < 0.2) that did not surpass FDR correction in any lineage (Table S13). These results are summarized in Fig. 4B, while UMAP feature plots (Fig. 4C) visualize the expression of CTSB and ECHDC3 across cell clusters.

### Stage 4: Structural and clinical impact of CTSB and ECHDC3

To assess the impact of CTSB on CSVD beyond WMH lesions, we also tested associations between each protein and other CSVD-related imaging and clinical traits: white matter microstructural integrity, enlarged perivascular spaces, and lacunar ischemic stroke^3^. In MR analyses, we found that CTSB had at least a moderate impact on these traits, with certain instruments showing more significant effects than others (Fig. 3, Table S6). In particular, we observed more pronounced effects of CTSB expression on enlarged perivascular spaces in the basal ganglia and hippocampus compared to the white matter location. For ECHDC3, the picture was much less clear, with most results being non-significant (Figure S2, Table S8). We observed a directionally consistent result for fractional anisotropy, a measure of white matter fiber organization. The reverse MR results were all null for both CTSB and ECHDC3 (Figures S3 and S4, Tables S7 and S9), providing no evidence that genetically determined CSVD traits causally influence circulating levels of either protein, and thereby arguing against reverse causation.

**Figure 5:**
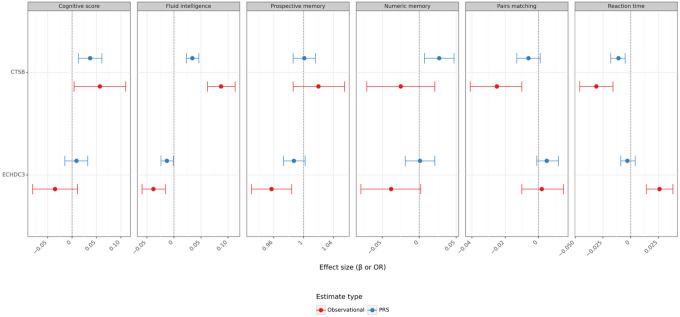
Associations of Protein Levels with Cognitive Performance. Forest plot comparing plasma-level and Polygenic Risk Score (PRS) estimates for associations of CTSB and ECHDC3 with six cognitive outcomes: general cognitive ability (defined as the first principal component of five touchscreen tests), fluid intelligence, prospective memory, numeric memory, pairs matching, and reaction time. Models were adjusted for age, sex, Townsend deprivation index, BMI, diabetes, hypertension, smoking status, and alcohol use. For pairs matching and reaction time, lower scores indicate better performance. Within each panel, points represent the effect estimate (β or odds ratio) and horizontal bars denote 95% confidence intervals. The dashed vertical line indicates the null effect (β = 0 for linear outcomes or an odds ratio of 1 for logistic outcomes). Source data are provided as a Source Data file.

Finally, we evaluated whether circulating or genetically determined levels of CTSB and ECHDC3 relate to cognitive performance in UK Biobank participants. The primary outcome was a previously validated measure of general cognitive ability derived as the first principal component of five touchscreen tests, following established transformations^21^. Higher plasma CTSB concentrations were associated with better general cognitive ability (β = 0.057 per 1 SD, 95% CI 0.004–0.11, p < 0.05) and better performance on four of the five component tasks, most notably fluid intelligence (β = 0.087, 95% CI 0.062–0.113, p < 1 × 10 ^10^) (Table S14). A cis-pQTL-based PRS for CTSB produced concordant estimates (β = 0.037, 95% CI 0.013–0.061, p = 0.002). By contrast, neither circulating nor genetically predicted ECHDC3 levels associated with the general cognitive ability score, although nominal links with poorer fluid intelligence and prospective memory were observed. The full pattern of observational and PRS results is shown in Fig. 5. Coupled with its lesion-level downregulation, our findings support a model in which CTSB confers resilience both structurally, by limiting WMH progression, and functionally, by preserving cognitive performance.

## DISCUSSION

We report the results of a multiomic study of WMH that integrates genomic, proteomic, single-nucleus transcriptomic, neuroimaging and clinical data from two different studies. Our study combines different analytical tools to isolate relevant pathways, increase scientific rigor, and provide evidence for causality. Leveraging data from more than 50,000 UK Biobank participants and eight post-mortem brains, we first demonstrate that higher plasma CTSB, as well as its cis-pQTL and cis-eQTL instruments, is associated with lower WMH burden. Single-nucleus RNA-seq then revealed marked lesion-specific downregulation of CTSB predominantly in astrocytes, indicating that local loss-of-function accompanies structural injury. Mendelian randomization analyses confirmed directionality and extended the results to related neuroimaging and clinical CSVD traits. These multi-layered data collectively nominate CTSB as a lesion-suppressed factor and plausible therapeutic entry point for mitigating WMH and preserving brain health.

One important limitation of WMH as a biomarker of CSVD is their biological heterogeneity, as they likely capture several different pathophysiological phenomena that end up with small vessel injury^8^. Our study was designed to overcome this biological heterogeneity, aiming to identify proteins and pathways that are hubs of the numerous contributors captured by polygenic effects. In addition, our conservative multistep analytical pipeline seeks to retain only those signals that remain significant in different stages each aimed at studying a specific biological step. While we recognize that several other proteins are likely to play a role in WMH emergence and evolution, those identified in this pipeline are highly likely to represent true candidates.

CTSB is a lysosomal protease that has not been fully characterized: lysosomal leakage in Alzheimer’s disease and traumatic brain injury models ties it to neuronal injury, yet exercise raises circulating CTSB and boosts hippocampal plasticity and memory^22,23^. Our findings linking CTSB to reduced WMH burden align with and extend previous research implicating lysosomal protease dysregulation in CSVD and neurodegeneration. This interpretation is directly concordant with the work of Lee et al.^24^, who found that higher levels of the endogenous CTSB inhibitor, cystatin C, predict faster WMH growth, consistent with our observation of a protective role for CTSB itself. Experimental models further demonstrate that lysosomal CTSB promotes oligodendrocyte autophagy, extracellular-matrix remodeling, and timely remyelination after demyelination injury^25–27^. Together, these concordant human and mechanistic data corroborate a reparative and protective role for CTSB and reinforce the consistency of our multi-layered observations.

Several reports appear to conflict with our findings, suggesting a harmful role of CTSB. In middle-cerebral-artery-occlusion models, genetic or pharmacological CTSB blockade reduces infarct volume by ≈ 30%^28,29^, suggesting deleterious actions in acute gray matter ischemia. Astrocytic CTSB release has also been shown to activate the NLRP3 inflammasome and exacerbate myelin damage under chronic hypoperfusion^30,31^. Importantly, these observations do not contradict our findings; rather, they underscore that CTSB’s net effect depends on disease acuity, lesion stage, and subcellular localization. Our data help reconcile these opposing views by considering the disease’s temporal dynamics. Post-injury profiling indicates a transient CTSB surge during the first 48 hours, followed by chronic suppression^28^; the early surge plausibly aligns with stroke models in which CTSB inhibition is beneficial, whereas WMH lesions capture the later phase in which CTSB is suppressed. Moreover, lysosomal CTSB promotes autophagic flux, whereas cytosolic leakage precipitates NLRP3 activation^31^. The lesional downregulation we observe therefore likely reflects loss of the protective lysosomal pool during chronic white matter injury rather than persistent harmful spill-over, coherently explaining why augmenting systemic CTSB correlates with white matter integrity while local depletion accompanies chronic damage.

Further supporting this interpretation, a recent proteogenomic study by Caro and colleagues independently identified CTSB among the proteins most robustly associated with WMH burden using Mendelian randomization across CSF and plasma, with directionally consistent results^32^. The two analyses are complementary: while Caro et al. employed a broad discovery framework spanning multiple cSVD phenotypes and biological fluids, our polygenic risk-anchored design is more restrictive by intent, enriching for proteins along the pathophysiological pathway from genomic predisposition to WMH. The identification of CTSB under these more stringent criteria helps prioritize it among their broader candidate set, and our single-nucleus transcriptomic analysis in periventricular white matter tissue provides disease-relevant cellular context that fluid-based biomarker approaches cannot directly offer.

In contrast to CTSB, the mitochondrial enzyme ECHDC3 failed to show a consistent or robust association with WMH or cognition beyond the initial polygenic risk screen in Stage 1. This null result is biologically plausible given ECHDC3’s known tissue-specificity, as it is an intracellular mitochondrial hydratase with low expression in the brain and a more prominent role in peripheral metabolic tissues like adipose^33–35^. Furthermore, ECHDC3’s influence on vascular health is likely indirect, operating upstream via systemic metabolic pathways such as insulin sensitivity^34^, and its effects may have been attenuated in our models by statistical adjustment for these same vascular risk factors. Given its modest genetic effect size^34^, detecting a significant association may require even larger cohorts. This null finding is therefore informative: it reinforces ECHDC3’s classification as an indirect metabolic modulator, thereby strengthening the specific case for CTSB as a more proximate and promising therapeutic target for CSVD. Given the conservative nature of our study design and analytical pipeline, we note that further research is needed to fully rule out ECHDC3 as possible target related to CSVD pathophysiology.

Several limitations must be considered when interpreting the results of our study. A primary limitation is that our findings are derived predominantly from the UK Biobank, a cohort of mainly European ancestry with a known “healthy volunteer” bias, which may limit the extrapolation of our effect sizes to more diverse or less healthy populations^36^. We must also acknowledge that circulating plasma protein levels may not perfectly mirror protein activity within the central nervous system and can be subject to batch effects and pre-analytical variability^37^. Additionally, our mechanistically informative lesion-level validation was based on a small number of post-mortem brain samples (N = 8), providing a cross-sectional snapshot that cannot capture the full dynamics of gene expression over time. Finally, automated WMH segmentation with BIANCA, used to centrally measure WMH in the UK Biobank, exhibits volume-dependent error and does not always accurately reflect true WMH volume^38^.

## CONCLUSION

By systematically integrating human proteogenomics in over 50,000 individuals with lesion-level transcriptomics, this study evaluated thousands of proteins as potential candidate factors in WMH pathology. Our multi-layered analysis identified CTSB as a lesion-suppressed yet systemically protective lysosomal protease, robustly associated with reduced WMH burden, related CSVD traits, and preserved cognitive function. Ultimately, these findings establish the regulated CTSB axis as a critical component of brain resilience against small vessel disease and nominate it as both a mechanistic target and a tractable therapeutic avenue, with potential to attenuate WMH progression and preserve cognition across the ageing brain.

## METHODS

### Ethics approval

This study was approved by the institutional review board of Yale University School of Medicine and adhered to the Declaration of Helsinki. All individuals gave written informed consent at baseline, and approval of the UKB study was obtained from the North West Multi-Center Research Ethics Committee (no. 11/NW/0382). Data were accessed using project application number 58743.

### Study design

We conducted a four-stage study (Fig. 1). In Stage 1, we sought to identify proteins that lie in the pathway between polygenic risk of white matter hyperintensities (WMH) and radiographic WMH volume. The criteria were adapted from previously described methods^15^ and included: (A) performing association analyses between a polygenic risk score of known WMH loci and 2,923 circulating protein levels, (B) evaluating proteins identified in step A for association with WMH volume (ascertained via research MRI, see below) and verifying concordance between the PRS–protein and protein–WMH associations. In Stage 2, we investigated the causality and effect directionality between the proteins identified in Stage 1 and WMH using bidirectional Mendelian Randomization analyses. In Stage 3, we studied the differential expression of identified proteins using single-nucleus RNA sequencing data obtained from four WMH lesions and four controls. Finally, in Stage 4, we extended our analyses beyond WMH and investigated the relationship between the identified proteins and related CSVD traits, as well as cognitive outcomes.

### Study participants

Participants were drawn from 2 different studies: the UK Biobank and the NIH NeuroBioBank (Request ID #1458) for the snRNA analyses. The UK Biobank is a population-based study of over 500,000 individuals aged 40 to 69, recruited between 2006 and 2010 from 22 centers across the United Kingdom^39^. Samples for genotyping and proteotyping assessments were collected at baseline. Beginning in 2014, a subset of 41,443 UK Biobank participants was selected for a neuroimaging substudy^40^, undergoing research brain MRI at a median of 9.3 years (Q1–Q3: 7.8–10.4) from baseline. From 2020 to 2021, the UKB-PPP (UKB Pharma Proteomics Project) analyzed 54,219 baseline blood samples for proteomic profiling of 2,923 proteins. A subset of 5,443 participants contributed data to both the proteomics and neuroimaging studies. The NIH NeuroBioBank is a network of brain banks across the United States that collects, stores, and distributes post-mortem human brain tissue for research purposes. For the single-nucleus RNA sequencing analyses, we obtained frozen post-mortem brain tissue from donors with pathologically confirmed WMH lesions and non-lesional white matter from the same individuals serving as internal controls.

### Proteomic data

Proteomic profiling was conducted on plasma samples obtained from UK Biobank participants, enabling the detection of 2,923 unique proteins. Details of these assays have been previously reported^41–43^, including comparisons with seven overlapping clinical assays, which showed strong agreement for matching isoforms (r = 0.82)^41^. In brief, Olink deploys proximity extension assays in which pairs of antibodies, each conjugated to complementary oligonucleotides, bind to the target proteins. Upon hybridization of the probes, amplification is performed, followed by relative quantification via next-generation sequencing. The protein-targeting assays are organized into four 384-plex panels corresponding to inflammation, oncology, cardiometabolic, and neurology. Olink incorporates multiple internal controls, including an incubation control (a nonhuman antigen with matched antibodies), an extension control (IgG conjugated with complementary oligonucleotides), and amplification controls (synthetic double-stranded DNA). Each plate also contains external controls, such as negative, plate, and sample controls. The limit of detection for each assay is computed per plate using triplicate runs of negative controls. Normalized protein expression (NPX) values are generated by normalizing to the extension control, applying a log2 transformation, and then performing an additional normalization step against the plate controls. Samples are flagged with a warning if NPX values for internal controls deviate from the plate median by more than ± 0.3 NPX within an abundance block or if the mean assay count for a sample falls below 500. Assays are similarly flagged if the median of the negative control triplicate deviates by over five standard deviations from Olink’s predefined values.

### Neuroimaging phenotypes

Participants selected for the neuroimaging substudy underwent high-quality MRI on a Siemens Skyra 3-Tesla scanner equipped with a standard 32-channel head coil. Multishell diffusion scans were acquired at two b-values (b = 1000 and 2000 s/mm^2), each with 50 distinct diffusion-encoding directions at 2 mm isotropic spatial resolution. The total diffusion scan duration was seven minutes^40^. In Stage 1, the primary outcome was WMH volume, segmented with the BIANCA algorithm by the UK Biobank^40,44^, then standardized for head size, natural-log transformed, and normalized. For Stage 2, we additionally evaluated fractional anisotropy (FA) and mean diffusivity (MD)—diffusion tensor imaging (DTI) measures of white matter integrity that, along with WMH, are validated predictors of stroke and dementia^7,45–47^. FA reflects the directional coherence of water diffusion, while MD characterizes the overall degree of water diffusion. FA and MD were estimated in 48 brain regions by the UKB using DTIFIT, an FSL^48^ tool for voxel-wise diffusion tensor modeling. To generate aggregate whole-brain measures, we used a previously described approach^40,49,50^, computing the first principal component across the 48 regions and subsequently applying normalization (as done for WMH). In secondary analyses, we examined FA and MD values of each region separately.

### Genetic data

A detailed description of DNA collection and genotyping is available elsewhere^51^. Briefly, DNA was extracted from blood samples collected at enrollment. Genotyping was performed by the Affymetrix Research Services Laboratory in 106 sequential batches of approximately 4,700 samples each. A subset of 49,950 participants enrolled in the UK Biobank Lung Exome Variant Evaluation study was genotyped using the Applied Biosystems UK BiLEVE Axiom Array, which contains 807,411 markers. Subsequently, 438,427 participants were genotyped using the closely related Applied Biosystems UK Biobank Axiom Array, containing 825,927 markers; 95% of these markers overlap with those on the UK BiLEVE Axiom Array. The UK Biobank research team applied standardized quality control for both arrays^51^, assessing batch effects, plate effects, departures from Hardy-Weinberg equilibrium, sex effects, array effects, and discordance across control replicates. Subject-level quality checks included tests for relatedness and heterozygosity, followed by principal component analysis for ancestry evaluation^52^. This pipeline produced 488,377 samples and 805,426 markers eligible for imputation, which was carried out using IMPUTE4^53^ with three reference panels (the Haplotype Reference Consortium^54^, UK10K haplotype^55^, and 1000 Genomes Phase 3^56^). The final imputed dataset included 93,095,623 autosomal SNPs, short indels, and large structural variants in 487,442 individuals, of whom 409,551 were of European ancestry and 77,891 were of other race/ethnic groups.

### Genetic analyses

We utilized genetic instruments derived from external genome-wide association studies to model polygenic predisposition and perform Mendelian Randomization.

#### PRS of WMH

To construct the PRS of WMH, we selected 27 independent single nucleotide polymorphisms (SNPs) identified in a large GWAS of WMH volume^16^. The identified loci account for effect modification or confounding by hypertension. This GWAS of mainly European ancestry was performed using 50,970 participants, including 26,788 from the UK Biobank. Given the small overlap between UKB participants included in both the imaging and proteomics sub-cohorts, we estimate that no more than 10% of the GWAS participants are also included in our Stage 1 analyses.

#### Protein pQTL

To derive instruments for our exposures and outcomes, we used protein Quantitative Trait Loci (pQTL) derived from the Iceland 36K study (deCODE). Our primary set of instruments comprised SNPs in cis association with the proteins (+/− 1Mb of the gene-coding region), genome-wide significant (p < 5×10^−8^) and uncorrelated (r^2^ < 0.001). If certain proteins identified in Stage 1 were not measured in the Iceland 36K study, we resorted to using the pQTL derived from the UKB-PPP cohort as instruments.

##### Brain eQTL:

Since the proteins considered could also be involved in the response of the brain tissues to the vascular injury observed in CSVD, we narrowed down the brain-specific effects of protein activity on WMH by leveraging eQTL derived from two brain tissue datasets: GTEx and the Psychencode study, which derived brain expression from 32,000 cells of major brain regions from 1,866 unique donors. As for the vascular eQTL, we combined lead cis-eQTL (within ± 1 Mb of the gene-coding region) that reached the nominal p-value threshold for the gene in any of the 13 brain tissues included in GTEx. If a variant was a lead variant in several brain tissues, we kept the association results corresponding to the lowest nominal p-value. The combined list of lead cis-eQTL was further pruned to ensure independence (r^2^ < 0.001) between the variants.

#### Vascular eQTL

Unlike pQTLs, which reflect circulating protein levels measured in plasma, eQTLs capture the gene mRNA expression levels directly in the tissue of interest. Since vascular disease is an important contributor to WMH, we considered instruments derived from 3 vascular tissues (tibial, carotid, and aortic arteries) and whole blood from the Genotype-Tissue Expression (GTEx) project, version 10. For each protein, we combined lead cis-eQTLs (within ± 1 Mb of the gene-coding region) that reached the nominal p-value threshold for the gene in any of the 4 vascular tissues included. If a variant was a lead variant in several tissues, we kept the association results corresponding to the lowest nominal p-value. The combined list of lead cis-eQTLs was further pruned to ensure independence (r^2^ < 0.001) between the variants. Additionally, we also considered instruments from the eQTLGen study, which leveraged blood-derived expression from 32,000 individuals.

#### CSVD-related traits

The summary statistics for FA (n = 17,663), and MD (n = 17,467) were obtained from Persyn et al^57^. The summary statistics for WMH (n = 50,970) were obtained from Sargurupremraj et al^16^. The summary statistics for enlarged perivascular spaces in the white matter (n = 9,607 cases, n = 30,215 controls), hippocampus (n = 9,339 cases, n = 30,756 controls), and basal ganglia (n = 9,189 cases, n = 30,811 controls) were obtained from Duperron et al^58^. The summary statistics for lacunar stroke (n = 6,030 cases, n = 219,389 controls) were obtained from Traylor et al^59^.

#### PRS of protein expression

To model the genetic effect of proteins identified in stage 1 on cognition, we constructed PRS based on cis-pQTL data from deCODE or UKB-PPP. The SNPs included in the PRS were in cis association with the proteins (+/− 1Mb of the gene-coding region), genome-wide significant (p < 5×10^−8^) and uncorrelated (r^2^ < 0.1).

### Cognitive assessment

The battery of cognitive tests administered by the UK Biobank was designed to be completed in an unsupervised fashion. Although study staff members were present in the clinic during the test administration, participants were expected to work through the cognitive assessments independently. A detailed description of the cognitive tests administered by the UK Biobank is available elsewhere^51^ and summarized in Table S15. Briefly, the cognitive assessments were designed to assess different cognitive domains in a short amount of time and to be completed on a touchscreen computer. At baseline, almost all participants completed the Pairs Memory test, a test of visual memory, and the Reaction Time test, a measure of processing speed. A portion of enrolled study participants also completed tests of working memory (Numeric Memory test), prospective memory (Prospective Memory), and verbal and numerical reasoning (Fluid Intelligence). Following prior reports,^60,61^ all five cognitive tests were entered into a principal components analysis after appropriate transformation of non-normally distributed metrics (Pairs Memory test values were log(x + 1)-transformed, and reaction time values were log-transformed). The normalized values of the first unrotated principal component were saved and used as general cognitive ability scores, where higher values represent better cognitive ability (i.e., increased speed and/or accuracy).

### PRS-Proteins-WMH associations

In Stage 1, the PRS of WMH (sum of dosages × weights) was standardized to have mean of 0 and standard deviation of 1 and tested for association with each of the 2,923 circulating protein levels (dependent variables) using linear regression. The regressions were adjusted for age, sex, and the first ten genetic principal components. The proteins that significantly associated with the PRS (p-value after FDR correction < 0.05) were further tested for association with WMH volume using linear regression. These regressions were adjusted for age, sex, and the first four genetic principal components.

### Mendelian Randomization

We used MR analyses to assess the causality and effect directionality between proteins selected during Stage 1 and several CSVD-related traits. Our primary MR analysis used the inverse variance weighted (IVW) method. We also implemented the weighted median method, a robust alternative to the IVW method that allows for up to 50% of the genetic variants used to be invalid instrumental variables without biasing the causal effect estimate^62^. Additionally, the weighted median approach is less sensitive to outliers than the IVW method, which can be useful in the presence of genetic variants with extreme effect estimates^63^. In addition, we applied the simple mode method, which groups all variant-specific causal estimates into their most densely populated cluster (the “mode”) and takes that value as the causal effect; this approach remains consistent so long as the largest subset of instruments with the same true effect outweighs any invalid variants^64^. We tested for horizontal pleiotropy (the possibility that the effect of the instrument on the outcome of interest is exerted through a pathway other than the exposure) using the Mendelian Randomization Pleiotropy Residual Sum and Outlier (MR-PRESSO^65^) global test with 10,000 simulations and the MR-Egger intercept term^66^. If horizontal pleiotropy was detected, we identified the responsible instruments using the MR-PRESSO outlier test and repeated the analyses after excluding them.

### Single-nucleus RNA analysis

Our single-nucleus RNA (snRNA) dataset contains data from 8 post-mortem samples of human periventricular white matter (PVWM) obtained from human brains. Samples were acquired through the NIH NeuroBioBank (Request ID #1458). Selected samples were matched for age and sex and limited to those with no or minimal neurofibrillary tangles (Braak & Braak stages 0, I or II (Table S10)). Nuclei extraction was performed as previously described^67^ and all steps were performed at 4°C. In brief, 50 mg of frozen periventricular white matter were mixed with 15 mL of homogenization buffer and Dounce-homogenized 10–15 times using the loose pestle and 10–15 times using the tight pestle (Wheaton Cat # 357544). The tissue suspension was then subjected to sucrose ultracentrifugation (Beckman Coulter Avanti JXN-26) at 24,000 rpm for 1 hr. Supernatant was discarded and nuclei were resuspended in chilled 1× PBS at a concentration of 1,000 nuclei/μL. Nuclei were then processed using Chromium Single Cell 3’ v3.1 technology (10X Genomics) and sequenced using a NovaSeq 6000 sequencer. Raw sequencing data were aligned to the human genome hg38 using Cell Ranger v7.0.0 with include-introns set to TRUE. Data were processed using Seurat v5. Several filters were used to exclude doublets and dying cells. Nuclei with unique feature counts over 6,000 or below 500 were removed as well as nuclei that contained higher than 5% mitochondrial counts. Data were scaled and normalized using SCTransform v2 and percentage of mitochondrial counts was regressed out using the vars.to.regress parameter. The first 50 principal components were calculated and used to integrate samples using the CCAIntegration method. Clustering was done using the Louvain algorithm and we generated UMAPs based on the first 40 principal components at a resolution of 0.1. Clusters were manually annotated based on their expression of previously validated cell type-specific markers^68–70^. We used Seurat’s Wilcoxon Rank Sum test to determine DEGs.

### Protein-Cognition associations

In Stage 4, we tested proteins identified in Stage 1 for associations with cognitive outcomes using linear or logistic regressions as appropriate. The primary outcome was a general cognitive ability score obtained as an aggregate metric of the 5 cognitive tests. The 5 cognitive tests were tested individually in secondary analyses. The models accounted for age, sex, Townsend deprivation index, BMI, diabetes, hypertension, smoking status, and alcohol use. As exposures, we used both the normalized plasma expression levels of the proteins as well as a polygenic risk score built from cis-pQTL SNPs.

## Supplementary Material

Supplementary Files

This is a list of supplementary files associated with this preprint. Click to download.

- CTSBWMHsupplementaryv5.0.docx
- CTSBSupplementaryTables.xlsx

## Data Availability

The data generated in this study are available in Supplementary Tables S1–S15. Source data for Figures 1–5 and Supplementary Figures S1–S5 are provided with this paper. All genetic instruments used are publicly available through the referenced publications.

## Code Availability

Observational analyses and plotting were performed using Python 3.9^71^. Genetic analyses including clumping, polygenic risk scoring and all Mendelian Randomization analyses were performed using the Genal 1.5^72^ package in Python 3.9.1^71^. The Genal package and associated code examples are publicly available at https://github.com/CypRiv/genal.
